# Stroke Characteristics and Outcomes of Adult Patients in Northwest Ethiopia

**DOI:** 10.3389/fneur.2020.00428

**Published:** 2020-05-19

**Authors:** Moges Baye, Amy Hintze, Chloe Gordon-Murer, Tatiana Mariscal, Gashaw Jember Belay, Aynishet Adane Gebremariam, Charmayne M. L. Hughes

**Affiliations:** ^1^Department of Physiotherapy, College of Medicine and Health Sciences, University of Gondar, Gondar, Ethiopia; ^2^Health Equity Institute, San Francisco State University, San Francisco, CA, United States; ^3^Department of Kinesiology, San Francisco State University, San Francisco, CA, United States; ^4^Department of Internal Medicine, School of Medicine, College of Medicine and Health Sciences, University of Gondar, Gondar, Ethiopia

**Keywords:** stroke, cerebrovascular accident, epidemiology, hospital, Ethiopia, medical record review

## Abstract

Stroke is becoming one of the leading causes of adult disability and death in sub-Saharan African countries. The aim of the present study is to provide an up-to-date account of the clinical and demographic characteristics of patients with stroke admitted to the University of Gondar College of Medicine and Health Science Comprehensive Specialized Hospital (CMHS). A hospital based retrospective study design was used to analyze the medical records of all patients with stroke admitted to CMHS from June 20th 2012 and April 30th 2018. Data were cleaned and entered into SPSS for analysis. Among the 448 patients with stroke admitted to CMHS, 58.0% (*n* = 260) of patients were female, and 42.0% (*n* = 188) were male. In the present sample, 141 (31.5%) had an ischemic stroke confirmed by neuroimaging, 82 (18.3%) had a hemorrhagic stroke confirmed by neuroimaging, and 252 (50.2%) had a stroke with undetermined type. The mean age of stroke was 63.9 years (range = 18–100, *SD*: 15.1 years), with no differences observed between stroke subtypes. The most common symptoms that led to patients seeking medical intervention were hemiparesis (67.4%), communication difficulties (56.0%), facial deviation (37.3%), and globalized headache (36.4%). Hypertension was the most commonly reported risk factor (37.1%), which was more prevalent in hemorrhagic (*n* = 37, 45.1%) than ischemic stroke patients (*n* = 53, 37.6%), stroke with undetermined type (*n* = 76, 33.8%). Stroke places a significant burden on sub-Saharan African countries. Results of the current study highlight the need to develop programs that educate the Ethiopian populace about the risk factors and symptoms of stroke, the importance of seeking medical care within the golden window, and the benefits of neuroimaging to accurately diagnose stroke subtype. In addition, the current study provides hospital administrators with empirical data that they can use to form an interdisciplinary stroke rehabilitation team capable of improving outcomes of Ethiopian patients with stroke.

## Introduction

Stroke is a leading cause of adult disability and death worldwide (1). Each year ~15 million people worldwide suffer a stroke, and five million of those are left with some type of permanent physical disability (2). Stroke is one of the leading causes of serious, long-term physical disabilities, and has pervasive negative influences on an individual's quality of life, participation in society, independence, emotions, and productivity (2, 3). Over the last four decades stroke prevalence in low- and middle-income countries has more than doubled, whereas the rate of stroke incidence in developed countries has decreased by 42% (1). In addition, low- and middle-income countries have some of the highest stroke mortality rates in the world, contributing to between 0.8 million and 5 million deaths annually (4). The growing corpus of research regarding the epidemiology of stroke in Ethiopia has indicated important differences in the clinical and demographic characteristics of patients compared to developed nations. Specifically, Ethiopian hospitals observe a higher percentage of hemorrhagic stroke compared to ischemic stroke (5, 6), which is much higher than that reported in developed nations (4). In addition, the mean age of stroke patients who present to Ethiopian hospitals [45.3 years (7) to 62.8 years (8)] is younger than stroke patients in Western countries [median age of 73 years, (9)].

While the aforementioned studies have provided valuable information on the epidemiology of stroke in Ethiopia, many of them have examined data from short time periods [e.g., 1 year, (5, 6)]. Only one study has examined stroke characteristics over a long time period (10). However, that study looked at data from 295 stroke patients at Tikur Anbassa Teaching Hospital between September 1990 and August 1996, and given the changes in lifestyle that are occurring in Africa, conducting an up-to-date assessment would afford the opportunity to determine if stroke characteristics in Ethiopia have evolved over time.

Motivated by this gap in the literature, the aim of the present study is to provide an up-to-date account of the clinical and demographic characteristics of individuals who presented with stroke to the University of Gondar College of Medicine and Health Science Comprehensive Specialized Hospital (CMHS) between June 20th 2012 and April 30th 2018. Results of this study will be used to inform the development of programs that educate the Ethiopian populace about the risk factors and symptoms of stroke, the importance of seeking medical care within the golden window, and the benefits of neuroimaging to accurately diagnose stroke subtype. In addition, data from the current study will be used by hospital administrators to form an interdisciplinary stroke rehabilitation team capable of improving outcomes of Ethiopian patients with stroke.

## Materials and Methods

### Study Design and Setting

A hospital based retrospective study design was used to analyze the medical records of all patients admitted to CMHS for stroke from June 20th 2012 and April 30th 2018. CMHS is one of the oldest hospitals in Ethiopia, providing a range of specialties, including pediatrics, surgery, gynecology, psychiatry, HIV care, and outpatient clinic rehabilitation care. The hospital is also one of the largest in Ethiopia, with more than 1,040 health care professionals, 580 beds in five inpatient departments and 14 wards, and outpatient services provided in 14 different units (11, 12), serving a population of outpatient and inpatient services for more than seven million individuals in the region (>450, 000 persons annually). As a referral teaching hospital, patient costs are less expensive than at non-teaching hospitals, and CMHS waives medical costs for individuals of low socioeconomic status. While there is no standalone stroke unit, CMHS has one neurosurgeon and two neurologists on staff, with nine hospital beds dedicated for individuals with stroke in the inpatient ward (although stroke patients may still occupy non-stroke specific beds when necessary). CMHS has a 4-slice CT scanner, with a current cost of 800 birr (~ $25 USD) for a brain scan. Due to its high cost [average base payment of $12,064, (13)], tissue plasminogen activator (tPA) is not available as a treatment for stroke.

**Figure 1:**
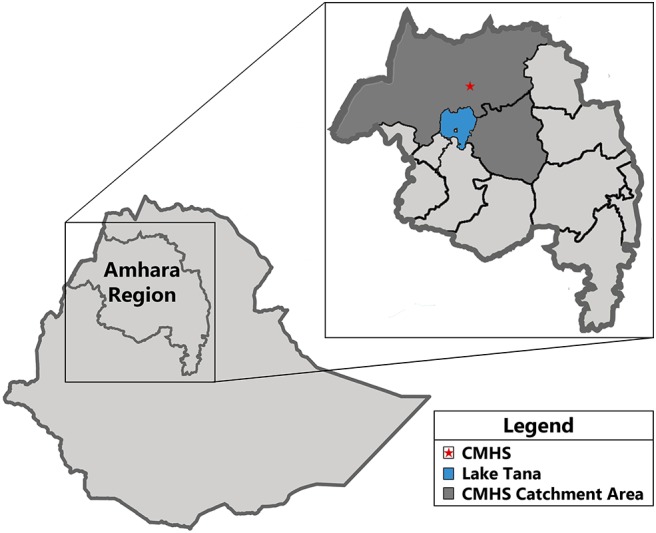
Map depicting the Amhara Region in Ethiopia, and the University of Gondar College of Medicine and Health Science Comprehensive Specialized Hospital catchment area.

Gondar is located in North West Ethiopia (Figure 1), ~748 km from the capital city (Addis Ababa) and 175 km from the Amhara state capital (Bahir Dar). Gondar has a long rainy season (known as Kiremt) that lasts from May 27 to September 25 (i.e., >49% chance of a given day being a wet day), with 143 days in the rainy season and 222 days in the dry season. Additionally, the CMHS catchment area has altitudinal variability ranging from 700 to 4,600 m (2,226 m in Gondar itself) above sea level, with average temperatures ranging from 16°C during the rainy season to 27°C in the dry season.

### Data Collection

Two reviewers examined the inpatient registration books to determine patients <18 who had been admitted with a diagnosis of stroke (HMIS admission and/or discharge disease classification with the keywords “stroke,” “hemiparesis,” “transient ischemic attack”) over the specified time period. Subsequently, trained data collectors examined the complete patient record located in the medical records office, and documented information about patients' demographics, risk factor profile, clinical presentation, stroke sub-type, vital signs upon hospital arrival, prescribed medications, whether brain scans were performed, and outcome at hospital discharge.

### Definition of Criteria

The World Health Organization's (WHO) definition of stroke as “a focal (or at times global) neurological impairment of sudden onset, and lasting ≥24 h (or leading to death), and of presumed vascular origin” (14) was used to provide the diagnosis of stroke. We used the following definitions for major stroke risk factors: hypertension (elevated blood pressure) was indicated by a blood pressure of 140/90 mm Hg or higher. Diabetes mellitus was indicated by fasting plasma glucose (FPG) test ≥7.0 mmol/l (126 mg/dl), a Glycated hemoglobin (HbA1c) level ≥6.5%, or a random plasma glucose (RPG) test ≥11.1 mmol/L (200 mg/dL) or previous diagnosis and treatment. Cardiac illness was defined by the presence of a cardiovascular condition such as coronary artery disease (CAD), myocardial infarction (MI), hypertensive heart disease, valvular heart disease (VHD), or congestive heart failure. Renal illness was defined by the presence of kidney diseases such as nephrosclerosis, acute kidney injury, chronic kidney disease, kidney stones, or kidney infections. Pulmonary illness was defined by the presence of chronic obstructive pulmonary disease (COPD), pneumonia, bronchitis, pleural effusion, or other disease that affects the lungs and other parts of the respiratory system. Tobacco smoking, alcohol intake, other drug use, family history of stroke, and mode of transportation to CMHS could not be reliably obtained from patient medical records, and as such was not included in analysis.

### Ethics Statement

The experiment was approved by the University of Gondar Institutional Review Board (IRB) and was conducted in accordance with the declaration of Helsinki.

### Data Analysis

Data were cleaned and entered into Statistical Package for the Social Sciences (SPSS, v27) for analysis. Statistics such as means, standard deviations, medians, and interquartile ranges were calculated to describe the typology of stroke, demographic characteristics, prevalence of risk factors, and treatment outcomes among stroke patients at CMHS. To compare differences between stroke subgroups, we used non-parametric tests (e.g., Mann Whitney *U*-Test, Kruskal-Wallis test, Chi-squared analysis) as appropriate. A probability value of ≤ 0.05 was considered significant.

## Results

A total of 665 potential patients with stroke were identified from the inpatient registration books. From these, 105 (15.8%) were missing from the medical records office or had incomplete patient records required to determine if they had a stroke, and 112 (16.7%) were found not to be stroke patients after reviewing their medical record. The age and sex of excluded participant records were not significantly different from included patient records (both *p's* > 0.05). Excluding these cases, there were 448 patient records fulfilling the WHO's criteria for stroke (14) based on clinical impression and/or neuroimaging. Stroke admissions comprised of 5.4% of all admissions to adult inpatient medical wards (excluding the orthopedic, maternity, and oncology wards).

Overall, 46.2% (*n* = 206) of patients had a CT scan and 3.8% (*n* = 17) had an MRI scan. Given that diagnostic certainty of stroke subtype requires neuroimaging, subsequent subtype analysis focused on three groups: ischemic stroke confirmed by neuroimaging (*n* = 141, 31.5%), hemorrhagic stroke confirmed by neuroimaging (*n* = 82, 18.3%), and stroke with undetermined type (*n* = 252, 50.2%).

### Demographics and Stroke Subtype

Of the 448 patients with stroke, 58.0% (*n* = 260) were female, and 42.0% (*n* = 188) were male. The mean age of stroke was 63.9 years (*SD* = 15.1, range = 18–100, median = 65.0, IQR = 55.0–75.0), with analysis revealing that stroke occurred at a slightly younger age for women (mean = 62.5, *SD* = 15.0, range = 18–95, median = 64.5, IQR = 54.8–75.0) compared to men (65.8, *SD* = 15.0, range = 25–100 years, median = 67.5, IQR = 58.0–78.0), *t*_(446)_ = −2.246, *p* = 0.025. The rate of stroke in young adults [defined as <50 years, (15)] was 15.0% (*n* = 68), with young stroke affecting women (*n* = 42, 9.4%) more than men (*n* = 25, 5.6%).

Results of Kruskal-Wallis analysis revealed that the mean age of stroke in the present sample was similar regardless of individuals had a hemorrhagic stroke (mean = 66.05, *SD* = 15.78, median = 69.5, IQR = 60.0–78.0), ischemic stroke (mean = 63.82, *SD* = 15.23, median = 65.0, IQR = 55.0–75.0), or stroke with undetermined type (mean = 63.12, *SD* = 14.66, median = 65.0, IQR = 55.0–75.0), H = 2.900, 2 df, *p* = 0.235. In addition, although the percentage of females was higher for both ischemic stroke (61.7%) and stroke with an undetermined type (59.1%), men and women had similar rates of hemorrhagic stroke (male = 51.2%, female = 48.8%). Despite these numerical differences, Chi-squared analysis revealed that the gender differences in this sample failed to reach significance, χ^2^ (1, N = 448) = 3.774, *p* = 0.152.

### Contextual Factors

**Figure 2:**
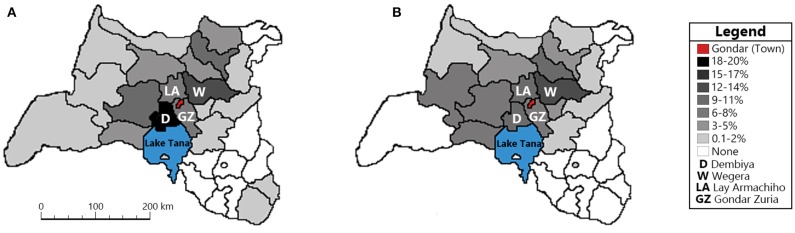
Map indicating the percentage of patients admitted to the University of Gondar College of Medicine and Health Science Comprehensive Specialized Hospital during the dry **(A)** and wet **(B)** seasons. Included are the four woredas closest to Gondar: Dembiya (D), Wegera (W), Lay Armachiho (LA), and Gondar Zuria (GZ). Patients admitted from the city of Gondar (37.9%) are not included.

Of the 448 patients, 37.9% (*n* = 170) lived in the city of Gondar. Of the 278 persons (62.1%) who lived outside Gondar, 91.4% came from woredas (regions) in North Gondar, 3.6% came from woredas in South Gondar, and 5.0% came from regions outside the hospital's catchment area (e.g., Kafta Humera, Bahir Dar). Regardless of the time of year, more people came to the hospital from the four woredas closest to the city of Gondar (Dembiya = 15.1% of 278, Wegera = 12.2% of 278, Lay Armachiho = 9.0% of 278, Gondar Zuria = 6.8% of 278, see Figure 2).

To determine whether there were differences in hospital admissions due to seasonality, we calculated the number of admissions per season (i.e., wet vs. dry season) for all years (i.e., 2012–2018). The time series data were then plotted and Augmented Dickey-Fuller test was used to examine series stability. Visits to CMHS were similar regardless of season (dry season = 17.2 visits, wet season = 18.1 visits), with the number of visits remaining relatively steady throughout the study period. The ADF test statistics (−4.996) were compared with the critical values from the non-standard Dickey–Fuller distribution with trend at the 5% significance level. Since the ADF statistic for the CMHS arrivals series is more negative than the critical value of −3.42, the null hypothesis of a unit root is rejected at the 5% level, implying that the time series was stationary.

### Admission Interval

The overall median time from symptom onset to hospital arrival was 48 h (IQR = 12.0–96.0). Hemorrhagic patients with stroke exhibited shorter onset-to-hospitalization times (median = 30.0, IQR = 13.0–72.0) than ischemic (median = 40.0, IQR = 12.0–120.0), and patients with an undetermined stroke type (median = 48, IQR = 13.0–96.0). When admission intervals where calculated based on whether patients reside within the city of Gondar or outside, analysis indicated shorter onset-to-hospitalization times for persons living in Gondar (median = 24.0, IQR = 7.0–72.0) compared to those who live outside Gondar (median = 48.0, IQR = 24.0–120.0). Cumulatively, 13.6% of patients from Gondar were admitted within 3 h of symptom onset, 25.3% within 6 h, and 37.7% within 12 h. More than 43.8% of patients from Gondar were admitted after 24 h, and 9.3% after 7 days. Symptom onset to hospitalization times were longer for patients residing outside of Gondar, with 4.2% of patients admitted within 3 h of symptom onset, 12.2% within 6 h, and 17.5% within 12 h. More than 59.8% of patients from outside Gondar were admitted after 24 h, and 16.1% after 7 days.

### Clinical History

Table 1 shows the clinical history of stroke patients separated by stroke subtype. Across all stroke subtypes, there were no substantial numerical differences in patients with a history of diabetes (*n* = 9, 2.0%), comorbid hypertension and diabetes (*n* = 22, 4.9%), cardiac illness (*n* = 34, 7.6%), and pulmonary illness (*n* = 22, 4.9%). In contrast, a clinical history of hypertension was the most commonly reported risk factor (*n* = 166, 37.1%), which was more prevalent in hemorrhagic (*n* = 37, 45.1%), than ischemic patients with stroke (*n* = 53, 37.6%) and patients with an undetermined stroke type (*n* = 76, 33.8%).

**Table 1:** Clinical history (not mutually exclusive) preceding hospitalization.

	**All patients** **(*n* = 448)**	**Ischemic** **(*n* = 141)**	**Hemorrhagic** **(*n* = 82)**	**Undetermined** **(225)**
HTN	166 (37.1%)	53 (37.6%)	37 (45.1%)	76 (33.8%)
NIDDM	8 (1.8%)	4 (2.8%)	2 (2.4%)	2 (0.9%)
Comorbid HTN & NIDDM	22 (4.9%)	6 (4.3%)	4 (4.9%)	12 (5.3%)
Cardiac illness	39 (8.7%)	14 (9.9%)	11 (13.4%)	14 (6.2%)
Pulmonary illness	13 (2.9%)	5 (3.5%)	3 (3.7%)	5 (2.2%)
Renal illness	11 (2.5%)	6 (4.3%)	2 (2.4%)	3 (1.3%)
Malaria	15 (3.3%)	2 (1.4%)	3 (3.7%)	10 (4.4%)
TB	14 (3.1%)	5 (3.5%)	2 (2.4%)	7 (3.1%)
HIV	8 (1.8%)	4 (2.8%)	0 (0.0%)	4 (1.8%)

*HTN, Hypertension; NIDDM, Non-insulin-dependent diabetes mellitus; TB, Tuberculosis; HIV, Human immunodeficiency virus*.

### Clinical Presentation

The majority of patients had highly elevated systolic blood pressure upon hospital arrival (*n* = 265, 59.2%), regardless of stroke subtype (ischemic = 56.7%, hemorrhagic = 64.6%, undetermined = 57.7%). In contrast, most patients presented with a heart rate within normal range (i.e., 60–100 bpm, *n* = 378, 84.4%). Analysis indicated that half of patients (ischemic = 51.4%, hemorrhagic = 47.6%, undetermined = 49.7%) presented with an elevated respiratory rate (i.e., 21–26/min) upon arrival at the hospital, 40.6% (ischemic = 43.3%, hemorrhagic = 42.7%, undetermined = 38.2%) presented with a normal respiratory rate (i.e., 12–20/min), and 9.4% with a respiratory rate >27/min (ischemic = 5.0%, hemorrhagic = 9.8%, undetermined = 12.0%). The majority of patients exhibited regular cardiac rhythm, with atrial fibrillation present in 16.1% of ischemic, 6.1% of hemorrhagic stroke patients, and 16.0% of stroke patients with an undetermined type.

As can be seen in Table 2, the most common symptoms that led to patients seeking medical intervention were hemiparesis (67.4%), communication difficulties (56.0%), and globalized headache (36.4%). Abnormal body movement (6.7%), incontinence (12.3%), hemiplegia (18.1%), and coma/altered mentation (19.6%) were reported in a small number of patients. Overall, ischemic patients with stroke were more likely to report hemiplegia (ischemic = 79.4%, hemorrhagic = 69.5%, undetermined = 59.1%), communication difficulties (ischemic = 60.3%, hemorrhagic = 50.0%, undetermined = 55.6%), and facial deviation symptoms (ischemic = 44.0%, hemorrhagic = 26.8%, undetermined = 36.9%) prior to hospital admission. However, analysis revealed that these differences were not significant, χ^2^ (4, N = 448) = 3.090, *p* = 0.543.

**Table 2:** Stroke symptoms and administered medications (not mutually exclusive).

	**All patients** **(*n* = 448)**	**Ischemic** **(*n* = 141)**	**Hemorrhagic** **(*n* = 82)**	**Undetermined** **(225)**
**STROKE SYMPTOMS**				
Hemiplegia	81 (18.1%)	15 (10.6%)	8 (9.8%)	58 (25.8%)
Hemiparesis	302 (67.4%)	112 (79.4%)	57 (69.5%)	133 (59.1%)
Abnormal body movement	30 (6.7%)	12 (8.5%)	6 (7.3%)	12 (5.3%)
Communication difficulties	251 (56.0%)	85 (60.3%)	41 (50.0%)	125 (55.6%)
Facial palsy	167 (37.3%)	62 (44.0%)	22 (26.8%)	83 (36.9%)
Coma or altered mentation	88 (19.6%)	31 (22.0%)	11 (13.4%)	46 (20.4%)
Repeated vomiting	117 (26.1%)	39 (27.7%)	18 (22.0%)	60 (26.7%)
Globalized headache	163 (36.4%)	53 (37.6%)	25 (30.5%)	85 (37.8%)
Incontinence	55 (12.3%)	14 (9.9%)	7 (8.5%)	34 (15.1%)
**ADMINISTERED MEDICATIONS**				
Aspirin	291 (65.0%)	96 (68.1%)	57 (69.5%)	138 (61.3%)
Statin	337 (75.2%)	110 (78.0%)	63 (76.8%)	164 (72.9%)
Antihypertensive	314 (70.1%)	106 (75.2%)	60 (73.2%)	148 (65.8%)
Anticoagulant	89 (19.9%)	26 (18.4%)	10 (12.2%)	53 (23.6%)
Antibiotic	78 (17.4%)	19 (13.5%)	16 (19.5%)	43 (19.1%)
Diuretic	37 (8.3%)	4 (2.8%)	8 (9.8%)	25 (11.0%)

*Statins, Atorvastatin (oral), Simvastatin (oral); antihypertensives, Amlodipine (oral), Enalapril (oral), Labetalol (oral), Anticoagulants, Warfarin (oral), Heparin (IV); Antibiotics, Azithromycin (oral), Metronidazole (IV); Ceftriaxone (IV), Gentamicin (IV); diuretic, Mannitol (IV), Metolazone (oral)*.

### Treatments

As can be seen in Table 2, the most commonly administered medications were statins (75.2%), aspirin (65.0%), and anti-hypertensive medications (70.1%). In contrast, <20% of patients were administered anti-coagulants (19.9%), antibiotics (17.4%), or diuretics (8.3%). More hemorrhagic and ischemic patients with stroke received anti-hypertensive medication (75.2% and 73.2%) than patients with a stroke with an undetermined type (65.8%), whereas anti-coagulants were more likely administered to patients with a stroke with an undetermined type (23.6%) than either ischemic or hemorrhagic patients with stroke (18.4 and 12.2%). However, results of Chi-squared analysis revealed that the differences in administered medications between groups was not statistically significant, χ^2^ (10, *N* = 448) = 6.346, *p* = 0.609. Of the 63 patients with atrial fibrillation, 31.7% (*n* = 20) received anticoagulants as part of medical treatment.

### Post-stroke Physical Outcomes

The most common post-stroke outcome was hemiparesis or hemiplegia, which affected 81.0% of participants in our sample. In comparison, aphasia affected 17.6% of patients (with more ischemic [19.9%] and undetermined stroke type [18.2%] exhibiting aphasia than hemorrhagic patients [12.2%]), while facial palsy affected 13.2% of patients. However, results of Chi-squared analysis revealed that the differences in outcome between groups was not statistically significant, χ^2^ (4, *N* = 448) = 3.635, *p* = 0.458.

The in-hospital fatality rate was 12.5% with 56 patient deaths. Among the 448 patients, 268 (59.8%) improved, 8 (1.8%) were referred to a higher facility (e.g., Tikur Anbessa Black Lion Hospital, Addis Ababa), 97 (21.7%) left the hospital against medical advice, and 17 (3.8%) absconded. Overall, the percentage of patients who improved was higher for ischemic and hemorrhagic subtypes (70.9 and 64.6%, respectively) compared to patients with stroke of an undetermined type (51.1%). In contrast, the number of patients who died was higher for stroke of an undetermined type (16.0%) compared to either ischemic or hemorrhagic subtypes (8.5 and 9.8%, respectively). Despite these numerical differences, Chi-squared analysis revealed that the differences in discharge were not statistically significant, χ^2^ (10, *N* = 448) = 14.20, *p* = 0.077.

The median duration of hospital stay was 9 days (mean = 10.9 days, range = 1–113 days, IQR = 5.0–15.0), with longer average hospital stays for patients with hemorrhagic stroke (median = 12.0, IQR = 7.0–16.8) than either ischemic (median = 10.0, IQR = 6.0–14.0) or undetermined stroke type (median = 8.0, IQR = 4.0–15.0). This difference was confirmed via Kruskal-Wallis test, H = 11.415, 2 df, *p* = 0.003. Overall, patients that improved stayed an average of 12.9 days (median = 11.0, IQR = 7.0–17.0), whereas patients who died stayed an average of 8.5 days (median = 5.0, IQR = 2.0–12.5), and those who absconded stayed for 11.9 days (median = 8.0, IQR = 2.75–13.75). In contrast, patients who left against medical advice stayed for 7.3 days (median = 5.0, IQR = 2.0–10.0), and those that were referred to a higher center stayed for 7.3 days (median = 6.0, IQR = 4.0–10.0).

## Discussion

The present study provides an up-to-date account of the clinical and demographic characteristics of patients with stroke who presented with stroke to CMHS between June 20th 2012 and April 30th 2018. In this study, ischemic stroke accounted for 31.5% of all stroke cases, hemorrhagic stroke accounted for 18.3% of all stroke cases, and patients with an undetermined stroke type accounted for 50.2% of stroke cases.

Novel to the current study was the examination of stroke admissions based on location and season. The catchment area of CMHS is extensive, encompassing individuals who reside in the city of Gondar, and individuals located as far as 300 km away. Regardless of the season (wet vs. dry), more than 50% of individuals resided in the city of Gondar or the four woredas closest to Gondar (i.e., Demiya, Wegera, Lay Armachiho, Gondar Zuria). Although the number of individuals from Gondar and the four closest woredas was lower during the rainy season, this reduction was more noticeable for patients coming from locations further away from CMHS. To understand why the number of individuals seeking medical services from CMHS for stroke decreases in the rainy season, one must consider the geography of the catchment area. It is characterized by rugged mountains, valleys, and gorges, with an elevation range between 700 m and 4,600 m (16), and receives between 1,100 and 1,360 mm of rainfall annually. As such, patients with mild stroke symptoms may not feel that going to the hospital is necessary and stay at home to see if their symptoms will resolve. For patients with stroke with moderate to severe hemiplegia/hemiparesis and/or altered mentation, this decision is compounded by the fact that many villages are located far from the main road, and thus they must first be carried across this terrain to the main road, and once there find a vehicle that will take them to the hospital.

Time plays a central role in acute stroke management, with early thrombolysis treatment minimizing damage to the brain and improving chances of recovery. Cumulatively, analysis indicated that only 13.6% of patients from Gondar were admitted within 3 h of symptom onset, with 37.7% admitted within 12 h of symptom onset. In contrast, cumulatively, only 4.2% of persons who live outside Gondar were admitted within 3 h, with 17.5% admitted within 12 h of symptom onset. Although the reasons that this sample of stroke persons did not present directly to hospital after the onset of stroke symptoms is unknown, prior research has indicating that the most common reasons for persons delaying medical intervention for stroke include not recognizing the symptoms as pertaining to stroke, not realizing the seriousness of the situation, and waiting to see if the symptoms would resolve themselves (17, 18). Moreover, it is also possible that Ethiopian patients may first seek help from religious or traditional healers, and only go to the hospital when their symptoms do not resolve. An alternative, and not mutually exclusive, explanation may relate to the size of the catchment area served by CMHS. Specifically, in addition to serving persons in townships within 10 km from the hospital (e.g., Azezo), individuals also travel across rugged terrain from far away townships such as Addi Arkay (179 km, ~ 3 h by car), Metema (196 km, ~ 3.5 h by car), Gayint (200 km, ~ 4.5 h by car), and Gelugu (279 km, ~ 5 h by car). As such, it may not be physically feasible for individuals from these latter townships to get to CMHS within the golden window, even if health education programs increased the awareness of stroke symptoms.

The other pertinent finding is that the prevalence of patients with a clinical history of hypertension (37.1%), diabetes (1.8%), or comorbid hypertension and diabetes (4.9%) is substantially lower than reported previously [cf. (19)]. The reduced prevalence of these two diseases in our sample is indeed intriguing, given the wealth of evidence indicating that hypertension and diabetes are two of the biggest risk factors for stroke (20, 21). The most likely reason for the decreased rates observed in the present study is that the many people reside in rural and deep-rural geographical regions and do not travel for regular medical care and check-ups. As such, they may be unaware of their chronic hypertensive and/or diabetic status, and were not clinically diagnosed with either until admission to CMHS. Because hypertension is the single most important risk factor of stroke, it is critical that efforts be made to develop and implement comprehensive preventative strategies to prevent, reduce, and control hypertension. One way to address the emerging hypertension burden in Ethiopia is to involve Health Extension Workers (i.e., members of a community trained to provide basic health and medical care to their community) in a lifestyle intervention that provides health promotion counseling on hypertension issues (e.g., increase physical activity, reduce salt and alcohol consumption, avoid tobacco and khat), and when needed, refers persons to the nearest health facility if the person has high blood pressure.

An unexpected finding to emerge from the current study is the high percentage of hemorrhagic patients with stroke (69.5%) and patients with stroke of an undetermined type (61.3%) that were prescribed aspirin during the course of in-hospital treatment. Although aspirin is effective in the early treatment of ischemic stroke, there is evidence that it may lead to hematoma enlargement, poorer outcomes, and increased risk of morbidity and mortality in hemorrhagic patients with stroke [cf. (22)]. However, in resource constrained environments, where barriers to timely CT scans delay clinical decision-making, physicians may balance the risks of aspirin administration against potential complications. For example, a cohort study of 148 Gambian patients with undetermined etiology (23) found that in-hospital aspirin use was associated with decreased mortality, despite the fact that 46% of patients were diagnosed to have had a hemorrhagic stroke. The use of aspirin in resource constrained environments is also supported by a recent study that evaluated the effect of aspirin administration on outcomes at hospital discharge using decision analysis (24). Results indicated that participants prescribed aspirin during initial hospitalization have an increased rate of stroke survival and a decrease rate of in-hospital stroke recurrence, compared to patients who do not have in-hospital aspirin treatment.

Our study also found that 81.1% of patients with stroke who sought medical treatment had hemiparesis or hemiplegia. Regaining lost limb function after a stroke requires extensive concentrated care from an interdisciplinary rehabilitation team, with inpatient rehabilitation consisting of at least 3 h of therapy 5 days per week (25), followed by 8 weeks of outpatient therapy 2–5 times per week (minimum of 45 min per day) where patients must travel to the clinic or hospital for treatment (26). At present, there are ~400 registered physiotherapists in Ethiopia to serve the estimated 15 million Ethiopians living with physical disabilities (27), with the majority of trained rehabilitation clinician working in urban areas despite the fact that 80% of Ethiopians live in rural areas (28) with limited access to health care services. Given the high number of patients presenting with hemiparesis and hemiplegia, as well as the lack of trained therapists in Ethiopia, the implementation of a context-appropriate tele-rehabilitation system (29, 30) could be one method by which patients with stroke could access rehabilitative care in their home environment, without the expense and logistical difficulties of constant travel to a hospital.

Another interesting finding to emerge from this study is the number of patients with stroke who exhibited post-stroke aphasia (17.6%) or facial palsy (13.2%). Clinical guidelines recommend that patients with aphasia have early access to a Speech-Language Pathologist (SLP) in order to facilitate the recovery of communication, assist in the development of compensatory strategies, and to involve family members in the therapy process (26). In contrast to physiotherapy, speech language therapy is still in its infancy in Ethiopia with the sole speech therapy center located at Yekatit 12 Hospital in Addis Ababa. However, the collaboration between the University of Toronto and the University of Addis Ababa has led to the establishment of the Bachelor of Speech-Language Pathology Degree, which will help support the many Ethiopian persons that suffer from post-stroke language disorders.

As with any study, there are a number of limitations that should be addressed in future work. First, patient medical records did not consistently include information regarding cigarette smoking, alcohol consumption, and a family history of stroke. The high level of missing data means that we are unable to speak to the prevalence of these modifiable risk factors in our sample, which are known to be key factors in the primary and secondary prevention of stroke (31, 32). Also, this level of missing data highlights the need for health care organizations to instantiate and standardize policies regarding the measurement and recording of patient and family health information. Second, despite the available evidence from Western countries indicating that 70% of patients with stroke exhibit impairments in the upper extremity 6-months post-stroke (33), and 83% of patients show cognitive impairments (34), we were unable to obtain information regarding the long-term outcomes from stroke in this population. Third, although CMHS has a 4-slice CT scanner, and patients with suspected stroke are advised to get a scan, half of patients in this sample elected not to have a CT scan. Thus, while the technology required to accurately diagnose stroke subtype is available, many patients may not be able to afford it, or it may take them too long to gather the money required for the procedure before acute stroke care begins. A lack of scan impairs the ability to administer the most appropriate treatment for the patient, leading to adverse health outcomes. This supposition is supposed by the data indicating that ischemic and hemorrhagic patients with stroke are more likely to exhibit improvements (70.9 and 64.6%, respectively), and are less likely to die (8.5 and 9.8%, respectively) compared to patients with stroke of an undetermined type (improve: 51.1%, die: 16.0%). Last, this was a retrospective hospital-based study. Thus, our data do not capture patients or experiencing a transient ischemic attack (TIA) whose symptoms may resolve themselves, or may not be deemed severe enough to necessitate travel to a tertiary care center, such as CMHS.

Despite these limitations, our study provided important information about the clinical and demographic characteristics of patients with stroke who are in the CHMS catchment area. Ethiopia is one of the poorest countries in the world, with an estimated 80–85% of the Ethiopian population living at, or below, US$0.50 per day (35). Although CMHS stroke care guidelines are based on data from high-income countries, stroke prevention and treatment is negatively impacted by the limited availability and large costs of thrombolytic therapy (e.g., tPA), the dearth of trained specialists, and the size of the CMHS catchment area. For example, standard care in high-income countries is that all admitted patients with suspected acute stroke receive a brain scan (e.g., CT) in order to make appropriate stroke management decisions (36), and that IV tPA be administered to eligible patients within 4.5 hours of hospital admission (37). The results of the current study (i.e., 13.6% of patients were admitted within the golden window, 50.2% of patients received a CT scan) indicate that Ethiopian public hospitals cannot rely solely on stroke care protocols established in high-income countries, highlighting the need to establish best practices for stroke care in low- and middle-income countries that take into consideration the financial constraints and a lack of infrastructure in these settings. As this work moves forward, the results of the current study will be used to develop programs that educate the Ethiopian populace about the risk factors and symptoms of stroke, the importance of seeking medical care within the golden window, and the benefits of neuroimaging to accurately diagnose stroke subtype. In addition, this study provides useful information by which hospital administrators can form an interdisciplinary stroke rehabilitation team capable of improving outcomes of Ethiopian patients with stroke.

## Data Availability Statement

The datasets generated for this study are available on request to the corresponding author.

## Ethics Statement

The studies involving human participants were reviewed and approved by University of Gondar Institutional Review Board. The ethics committee waived the requirement of written informed consent for participation.

## Author Contributions

CH, MB, and TM designed the experiment and formulated the experimental question. CH, TM, CG-M, MB, GB, and AG collected the data. CH, CG-M, and AH performed the data analysis and statistics. CH, CG-M, MB, GB, AH, and AG wrote the paper. All authors critically revised the manuscript and have approved the final manuscript.

## Conflict of Interest

The authors declare that the research was conducted in the absence of any commercial or financial relationships that could be construed as a potential conflict of interest.

## References

[1] Johnson W Onuma O Owolabi M Sachdev S. Stroke: a global response is needed. Bull World Health Organ. (2016) 94:634–4. 10.2471/BLT.16.181636 27708464 PMC5034645

[2] Go AS Mozaffarian D Roger VL Benjamin EJ Berry JD Blaha MJ Heart disease and stroke statistics - 2014 update. Circulation. (2014) 129:143–52. 10.1161/01.cir.0000441139.02102.80 PMC5408159 24352519

[3] Akinpelu AO Gbiri C. Quality of life of stroke survivors and apparently healthy individuals in southwestern Nigeria. Physiother Theory Pract. (2009) 25:14–20. 10.1080/09593980802622669 19140078

[4] Feigin VL Nichols E Alam T Bannick MS Beghi E Blake N Global, regional, and national burden of neurological disorders, 1990–2016: a systematic analysis for the Global Burden of Disease Study 2016. Lancet Neurol. (2019) 18:459–80. 10.1016/S1474-4422(18)30499-X 30879893 PMC6459001

[5] Alemayehu CM Birhanesilasie SK Assessment of stroke patients: occurrence of unusually high number of haemorrhagic stroke cases in Tikur Anbassa Specialized Hospital, Addis Ababa, Ethiopia. Clin Med Res. (2013) 2:94–100. 10.11648/j.cmr.20130205.11

[6] Deresse B Shaweno D. Epidemiology and in-hospital outcome of stroke in South Ethiopia. J Neur Sci. (2015) 355:138–42. 10.1016/j.jns.2015.06.001 26059446

[7] Tafessse A Subarachnoid hemorrhage: clinical presentation, causes and outcome in 52 Ethiopian patients. Ethiop Med J. (2018) 56:2.

[8] Gebremariam SA Yang HS. Types, risk profiles, and outcomes of stroke patients in a tertiary teaching hospital in northern Ethiopia. eNeuroSci. (2016) 3:41–7. 10.1016/j.ensci.2016.02.010 29430535 PMC5803092

[9] Heuschmann PU Wiedmann S Wellwood I Rudd A Di Carlo A Bejot Y. Three-month stroke outcome: the European Registers of Stroke (EROS) investigators. Neurol. (2011) 76:159–65. 10.1212/WNL.0b013e318206ca1e 21148118

[10] Alemayehu B Oli K Stroke admission to Tikur Anbassa Teaching Hospital: with emphasis on stroke in the young. Ethiop J Health Dev. (2002) 16:309–15. 10.4314/ejhd.v16i3.9799

[11] Gedif G Sisay Y Alebel A Belay YA Level of job satisfaction and associated factors among health care professionals working at University of Gondar Referral Hospital, Northwest Ethiopia: a cross-sectional study. BMC Res Notes. (2018) 11:824 10.1186/s13104-018-3918-0 30458846 PMC6245915

[12] University of Gondar Comprehensive Specialized Hospital. Planning, Monitoring and Evaluation Department Report (2017) Gondar.

[13] Kleindorfer D Broderick J Demaerschalk B Saver J. Cost of Alteplase has more than doubled over the past decade. Stroke. (2017) 48:2000–2. 10.1161/STROKEAHA.116.015822 28536176

[14] World Health Organization Noncommunicable Diseases and Mental Health Cluster. WHO STEPS stroke manual: the WHO STEPwise approach to stroke surveillance / Noncommunicable Diseases and Mental Health, World Health Organization. World Health Organization (2005). Available online at: https://apps.who.int/iris/handle/10665/43420

[15] Yesilot Barlas N Putaala J Waje-Andreassen U Vassilopoulou S Nardi K Odier C. Etiology of first-ever ischaemic stroke in European young adults: the 15 cities young stroke study. Eur J Neurol. (2013) 20:1431–9. 10.1111/ene.12228 23837733

[16] Ayalew D Tesfaye K Mamo G Yitaferu B Bayu W Variability of rainfall and its current trend in Amhara region, Ethiopia. Afr J Agric Res. (2012) 7:1475–86. 10.5897/AJAR11.698

[17] Moser DK Kimble LP Alberts MJ Alonzo A Croft JB Dracup K. Reducing delay in seeking treatment by patients with acute coronary syndrome and stroke: a scientific statement from the American Heart Association Council on cardiovascular nursing and stroke council. Circulation. (2006) 114:168–82. 10.1161/CIRCULATIONAHA.106.176040 16801458

[18] Schroeder EB Rosamond WD Morris DL Evenson KR Hinn AR Determinants of use of emergency medical services in a population with stroke symptoms. The second delays in accessing stroke health-care (DASH II) study. Stroke. (2000) 31:2591–6. 10.1161/01.STR.31.11.2591 11062280

[19] O'Donnell MJ Xavier D Liu L Zhang H Chin SL Rao-Melacini P. Risk factors for ischaemic and intracerebral haemorrhagic stroke in 22 countries (the INTERSTROKE study): a case-control study. Lancet. (2010) 376:112–23. 10.1016/S0140-6736(10)60834-3 20561675

[20] Mensah GA. Epidemiology of stroke and high blood pressure in Africa. Heart. (2008) 94:697–705. 18308869 10.1136/hrt.2007.127753

[21] Walker RW McLarty DG Kitange HM Whiting D Masuki G Mtasiwa DM Machibya H Unwin N Alberti KM. Stroke mortality in urban and rural Tanzania. The Lancet. (2000). 355:1684–1687. 10905244 10.1016/s0140-6736(00)02240-6

[22] Behrouz R Miller CM. Aspirin and intracerebral hemorrhage: where are we now? Neur Clin Pract. (2015) 5:11–6. 10.1212/CPJ.0000000000000089 29443177 PMC5764423

[23] Garbusinski JM van der Sande MA Bartholome EJ Dramaix M Gaye A Coleman R. *Stroke* presentation and outcome in developing countries: a prospective study in the Gambia. Stroke. (2005) 36:1388–93. 10.1161/01.STR.0000170717.91591.7d 15947255

[24] Berkowitz A Westover M Bianchi M Chou H. Aspirin for acute stroke of unknown etiology in resource-limited settings: a decision analysis. Neurology. (2014) 83:787–93. 10.1212/WNL.0000000000000730 25056582 PMC4155044

[25] Burris JE. Stroke rehabilitation: current american stroke association guidelines, care, and implications for practice. Missouri Med. (2017) 114:40. 30233099 PMC6143585

[26] Hebert D Lindsay MP McIntyre A Kirton A Rumney PG Bagg S. Canadian stroke best practice recommendations: stroke rehabilitation practice guidelines, update 2015. Int J Stroke. (2016) 11:459–84. 10.1177/1747493016643553 27079654

[27] World Health Organization World Bank. World Report on Disability. Geneva: World Health Organ (2011).

[28] Spielman DJ Davis K Negash M Ayele G Rural innovation systems and networks: findings from a study of Ethiopian smallholders. Agricult Hum Values. (2011) 28:195–212. 10.1007/s10460-010-9273-y

[29] Hughes CML Louie A Sun S Gordon-Murer C Belay G Baye M. Development of a post-stroke upper limb rehabilitation wearable sensor for use in sub-Saharan Africa. Front Bioeng Biotech. (2019) 7:322. 10.3389/fbioe.2019.00322 31781556 PMC6861447

[30] Hughes CML Padilla A Hintze A Mariscal T Sera M Weidner S Developing an mhealth app for post-stroke upper limb rehabilitation: feedback from U.S. and Ethiopian rehabilitation clinicians. Health Inform J. (2019) 460458219868356. 10.2196/preprints.12706 31566456

[31] Chung JW Kim BJ Han MK Kang K Park JM Park SS. Family history and risk of recurrent stroke. Stroke. (2016) 47:1990–6. 10.1161/STROKEAHA.116.013148 27406105

[32] Paciaroni M Bogousslavsky J. Primary and secondary prevention of ischemic stroke. Eur Neurol. (2010) 63:267–78. 10.1159/000285183 20357456

[33] Lawrence ES Coshall C Dundas R Stewart J Rudd AG Howard R. Estimates of the prevalence of acute stroke impairments and disability in a multiethnic population. Stroke. (2001) 32:1279–84. 10.1161/01.STR.32.6.1279 11387487

[34] Jokinen H Melkas S Ylikoski R Pohjasvaara T Kaste M Erkinjuntti T. Post-stroke cognitive impairment is common even after successful clinical recovery. Eur J Neurol. (2015) 22:1288–94. 10.1111/ene.12743 26040251

[35] Farm Africa Our Work in Ethiopia. (2018). Available online at: https://www.farmafrica.org/us/ethiopia/ethiopia (accessed December 5, 2018).

[36] Berkowitz AL. Managing acute stroke in low-resource settings. Bull World Health Organ. (2016) 94:554. 10.2471/BLT.15.162610 27429496 PMC4933138

[37] Powers WJ Rabinstein AA Ackerson T Adeoye OM Bambakidis NC Becker K. 2018 guidelines for the early management of patients with acute ischemic stroke: a guideline for healthcare professionals from the American Heart Association/American Stroke Association. Stroke. (2018) 49:e46–99. 10.1161/STR.0000000000000158 29367334

[38] Patil RR. Urbanization as a determinant of health: a socioepidemiological perspective. Soc Work Public Health. (2014) 29:335–41. 10.1080/19371918.2013.821360 24871771

[39] Gudina EK Michael Y Assegid S. Prevalence of hypertension and its risk factors in southwest Ethiopia: a hospital-based cross-sectional survey. Integ Blood Press Control. (2013) 6:111–7. 10.2147/IBPC.S47298 23986649 PMC3753877

